# Multiple wh-interrogatives in child heritage Romanian: On-line comprehension and production

**DOI:** 10.3389/fpsyg.2022.1018225

**Published:** 2022-11-22

**Authors:** Anamaria Bentea, Theodoros Marinis

**Affiliations:** ^1^Department of Linguistics, University of Konstanz, Konstanz, Germany; ^2^School of Psychology and Clinical Language Sciences, University of Reading, Reading, United Kingdom

**Keywords:** heritage language, child bilingualism, multiple interrogatives, Romanian, self-paced listening, elicited production

## Abstract

This study compared the online comprehension and the production of multiple interrogatives in 18 Romanian-English bilingual children aged 6;0–9;2 (*M_AGE_* = 8;0) living in the UK who have Romanian as heritage language (L1) and English as majority language (L2) and 32 Romanian monolingual children aged 6;11 to 9;8 (*M_AGE_* = 8;3). We examined whether differences emerge between heritage and monolingual children in the online comprehension and in the production of multiple interrogatives in Romanian, which requires fronting of all wh-phrases, contrary to English. The main aim was to uncover to which extent similarities or differences in morphosyntactic properties between the L1 and the L2 systems affect the acquisition and processing of the heritage language/L1. Online comprehension was assessed in a self-paced listening task, while production was assessed using an elicitation task. The results reveal that Romanian heritage children show similar online comprehension patterns to monolingual children for multiple interrogatives in Romanian. A different pattern emerges for production as heritage children produce less complex multiple questions in Romanian and avoid movement of two wh-phrases in all elicited structures. Given that their predominant responses for multiple interrogatives only make use of the structural option present in English, namely one fronted wh-phrase and one *in-situ*, we take this to show that there is transfer from the majority language to the heritage language. Thus, language production in the children’s L1 seems to be affected by properties of the dominant L2, under cross-linguistic influence. Taken together, the results for both comprehension and production suggest that heritage children are able to establish the underlying representation of multiple wh-movement structures, similarly to monolinguals, but have difficulties activating the more complex structure in production.

## Introduction

Various studies on heritage language (HL) acquisition have investigated the end-state grammars of adult HL speakers (Montrul, 2016; Polinsky, 2018; Polinsky and Scontras, 2020a,b) and have shown that they are highly heterogeneous in terms of first language (L1) acquisition outcomes and typically diverge from monolinguals in their L1 when tested in offline comprehension and production (Benmamoun et al., 2013; Montrul, 2016; Polinsky and Scontras, 2020a). This variability resembles that often found among second language (L2) learners, although L1 exposure starts from birth (Kupisch and Rothman, 2018). In contrast, few studies have focused on exploring how HL grammatical knowledge is accessed and implemented during on-line language processing (see Bayram et al., 2021; Jegerski and Sekerina, 2021 for a review) and even less is known about online language processing in HL children, children who speak a language that is different from the dominant societal language (Kupisch and Rothman, 2018).

The present study aims to bring further insights into HL development in child heritage speakers by comparing the performance of Romanian heritage children with L2 English to L1 Romanian-speaking children raised monolingually using both on-line comprehension and production tasks. In order to better understand how differences in surface syntactic structure between the heritage and the dominant societal language affect HL development, we examined whether heritage children pattern similarly to monolingual children on the real-time processing and the production of various types of multiple wh-questions, which display different syntactic properties in the heritage language, Romanian, and in the societal language, English: while Romanian fronts both wh-words, English, only fronts one wh-word, the second one remaining *in situ*. By investigating performance under different modalities, we aimed to get a more straightforward glimpse at the nature of the differences between child heritage speakers and child monolingual L1 speakers and how this relates to cross-linguistic influence (Serratrice, 2013; Meir and Janssen, 2021; van Dijk et al., 2021).

The paper is organized as follows. We first review previous studies on cross-linguistic influence in bilingual children with a focus on HL development. Then we present the properties of multiple wh-questions in Romanian and the findings for the acquisition of these structures in monolingual children. We conclude the introductory section with the research questions and predictions of the current study. We proceed with the presentation of participants, methods, and procedure. We then present the results, followed by discussion and conclusion.

### Cross-linguistic influence in early bilingual acquisition

The topic of cross-linguistic influence at the level of morphosyntax has been extensively investigated in child bilingualism (see Serratrice (2013) for an overview and van Dijk et al. (2021) for a recent meta-analysis evaluating cross-linguistic influence across 26 experimental studies). Research has shown that one language can have an effect on the other language at a morphosyntactic level (Hulk and Müller, 2000) and can lead to differences between monolingual and bilingual children which can be either quantitative, qualitative, or both. Quantitative differences stem from the frequency with which a certain structure is accepted or used by bilingual compared to monolingual children (Serratrice et al., 2004; Argyri and Sorace, 2007; Nicoladis and Gavrila, 2015). In other words, a phenomenon also present in monolingual development is reinforced in bilingual development under the influence of one language over the other. Qualitative differences stem from the presence of different language patterns in bilingual children’s production and comprehension relative to monolinguals (Nicoladis, 2006, 2012; Strik and Pérez-Leroux, 2011). Recently, Bosch and Unsworth (2020) investigated cross-linguistic influence in the production and acceptability of V2 word orders in English-Dutch bilingual children and found both quantitative and qualitative differences. Bilinguals accepted V2 orders with auxiliary verbs significantly more than monolingual children, but also accepted V2 with main verbs, contrary to monolinguals.

According to Hulk and Müller (2000) and Müller and Hulk (2001), cross-linguistic influence holds when the child’s two languages overlap at the surface level. If one language (language A) displays two structural options and the other language (language B) only makes one of these options available, then the option shared by the two languages may be reinforced in language A under influence from language B. In other words, “*there has to be a certain overlap of the two systems at the surface level*” (Hulk and Müller, 2000, p. 228–229). However, there is mixed evidence from the literature showing that cross-linguistic influence does not hold even in the presence of such structural overlap (Argyri and Sorace, 2007) or that cross-linguistic influence occurs in the absence of structural overlap (Nicoladis, 2006, 2012). Importantly, cross-linguistic influence does not seem to occur all the time and one of the factors that has been proposed to influence cross-linguistic influence is language dominance, which refers to the language that the child uses more frequently or the language in which the child has higher proficiency (Yip and Matthews, 2006). Here the prediction is that cross-linguistic influence goes from children’s dominant language into their weaker language (van Dijk et al., 2021), although there are also studies which found no relation between cross-linguistic influence and language dominance (Blom, 2010; Serratrice et al., 2012), showing that cross-linguistic influence can occur independently of language dominance.

While the majority of studies on early bilingual acquisition has investigated children’s offline comprehension, judgements, and production, only a few have examined real-time sentence processing in bilingual children. These have mainly focused on early L2 learners and compared children’s real-time processing of L2 morphosyntactic properties to that of their monolingual peers (Marinis, 2007; Chondrogianni and Marinis, 2012; Marinis and Saddy, 2013; Chondrogianni et al., 2015a,b) and generally report qualitatively similar processing patterns in bilinguals and monolinguals. Lemmerth and Hopp (2019) and van Dijk et al. (2022) specifically tested the effects of cross-linguistic influence on bilingual children’s on-line sentence processing. van Dijk et al. (2022), for example, tested English-Dutch and German-Dutch bilinguals aged 5 to 9 on a self-paced listening task assessing processing of word order in Dutch sentences. They found similar listening patterns in the V2 and V3 condition in Dutch in both monolinguals and bilinguals, but also report effects of cross-linguistic influence in the German-Dutch group in the condition instantiating a structural overlap between the two languages. In other words, the German-Dutch bilinguals slowed down when listening to V2 structures in Dutch and this slowdown was more pronounced in children who were more German dominant.

In contrast to the substantial literature on L2 acquisition, comparatively fewer studies investigated the acquisition of morphosyntax in HL development and how this is affected by cross-linguistic influence from the societal language. Some studies found no effects of cross-linguistic, suggesting that language-external factors shape child HL development (Daskalaki et al., 2019; Rodina et al., 2020). Other studies linked the differences in performance between child heritage speakers and monolinguals to the properties of the societal language (Meir et al., 2017; Meir and Janssen, 2021).

The acquisition of wh-dependencies in the HL has also received little attention. Cuza (2016) used an elicited production task to assess subject-verb inversion in matrix and embedded questions in Spanish heritage children aged 5;0 to 13;3 born and raised in the US. The results showed that Spanish-English bilingual children produce subject-verb inversion in Spanish to a significantly lower rate that their monolingual peers and that they also use subject-verb inversion less in embedded compared to matrix questions. Cuza (2016) argues that this pattern of performance arises from the interplay between cross-linguistic influence from English, the societal language, language dominance and issues of structural complexity. In a similar vein, Strik and Pérez-Leroux (2011) assessed Dutch-French bilinguals aged 5 to 7 and living in France on the production of wh-questions in Dutch, their L1. Although Strik and Pérez-Leroux (2011) do not use the label Dutch heritage speaker for their bilingual group, the children included in their study match the criteria used to define HSs (see Kupisch and Rothman (2018) for a discussion on HL terminology and early child bilingualism). Strik and Pérez-Leroux (2011) found that some of the wh-questions that bilingual children produced in Dutch differed qualitatively from those produced by Dutch monolingual children and followed a French-like structure. These were questions with a fronted wh-phrase and without subject-verb inversion, like *Wat jij doe giraffe? (lit. What you do giraffe?), and also wh-*in-situ* questions as in *Jij doe wat giraffe? (lit. You do what giraffe?). According to Strik and Pérez-Leroux, complexity is a trigger for cross-linguistic influence such that structures involving less derivational complexity in one language (e.g., *in-situ* questions) may impact structures which are derivationally more complex in the other language (e.g., wh-fronting with subject-verb inversion).

These previous works reporting different performance patterns in heritage compared to monolingual children assessed only children’s productive skills in their heritage language/L1. Various studies with monolinguals and bilinguals have revealed asymmetries between comprehension and production (Hendriks and Koster, 2010; Grimm et al., 2011) and although there are studies showing that production outpaces comprehension (see Hendriks (2014), Martinez-Nieto and Restrepo (2022) for the acquisition of pronouns), other studies report better performance in comprehension compared to production. Chondrogianni and Marinis (2012), for example, examined the on-line processing and production of tense and non-tense morphemes in L2 English children and children with Developmental Language Disorder (DLD). While the DLD children manifested difficulties with both comprehension and production, the typically-developing L2 children showed on-line sensitivity to the omission of tense morphemes, similarly to the L1 English children, despite variable production rates. Haiden et al. (2009) compared the comprehension and production of wh-questions in French by English- speaking children with L2 French and found high accuracy rates for their comprehension of questions with wh-fronting, on a par with those. In this study we compare HL children’s production to their real-time comprehension of multiple wh-questions and use both off-line and on-line methods. This can reveal whether HL children show qualitatively similar processing patterns to monolinguals but also whether asymmetries appear in the comprehension and production of questions with multiple wh-movement.

### Multiple wh-interrogatives in (child) Romanian

Full acquisition of multiple wh-questions involves various aspects that are subject to cross-linguistic variation. We will briefly outline the properties of multiple wh-questions that Romanian-speaking children need to acquire, by putting emphasis on differences with English. (1) illustrates multiple *who*-questions and (2) exemplifies *which-*questions in Romanian.

1.a.  Cine    pe cine  acoperă?
      who.Nom PE who  covers
      ‘Who is covering whom?’
   b.  *Pe cine  cine  acoperă?
      PE who  who covers
      *‘Whom is covering who?’
  2.a.  Care  fată  pe care  băiat_j_ îl_j_   acoperă?
      which girl  PE which boy_j_    him_j_  covers
      ‘Which girl is covering which boy?’
    b.  Pe care  băiat_j_ care  fată  îl_j_   acoperă?
      PE which  boy_j_  which girl him_j_  covers
      ‘Which boy is which girl covering?’

In terms of **lexical properties** of wh-words, wh-objects in Romanian are marked with a differential object marker *pe*, similar to *a* in Spanish. Although in the prescriptive use of English, *who* shows overt case-assignment in the form of *whom*, increasingly native English-speakers use *who* instead in informal spoken contexts (Aarts, 1994). Additionally, *care (‘which’)*-phrases in Romanian are doubled by a co-indexed clitic pronoun *îl* (‘him’) for masculine and *o* (‘her’) for feminine.

In terms of **movement properties**, wh-words move overtly, the difference with respect to English being that multiple wh-words in Romanian move together to a clause-initial position, as shown by (1) and (2) above. This is a property that Romanian shares with Bulgarian and other Slavic languages. According to Alboiu (2002), multiple wh-constructions in Romanian are derived by first moving the closest candidate (the subject), defined in terms of c-command, to a Spec,XP position. The remaining phrases then move *via* a ‘tucking in’ mechanism (see Richards, 1997) below the specifier created by the moved subject and this ‘tucking in’ movement of the following wh-phrases can take place in any order. On the other hand, fronting a *who-*object over a *who-*subject is ungrammatical (in both Romanian and English), as indicated by the asterisk in example (1b). Movement of wh-words in both languages obeys Superiority (Chomsky, 1973), a condition that limits the ordering of wh-words and blocks one wh-word from moving over another wh-word occupying a hierarchically higher position in the structure. Alboiu (2002) suggests that Superiority is observed in Romanian under her proposed analysis. Given that the subject occupies a structurally higher position and is the closest candidate, it should move first. This requirement does not hold for *which*-questions, as evidenced by the grammaticality of the example in (2b; see Pesetsky (2000) for an explanation). Laenzlinger and Soare (2005) and Soare (2009) convincingly argue for Romanian that *which*-expressions always appear clause-initially, preceding *who*-phrases. By adopting a split-CP analysis (Rizzi, 1997) and a cartographic approach to syntactic structures (Rizzi, 2004), Soare (2009) shows that *which-*phrases target the specifier position of a Topic head above the specifier Focus position which they postulate as the landing site of *who*-phrases.

The **semantic properties** of multiple wh-questions require establishing a pairing relation between the wh-phrases: a felicitous answer for a question like (1a) is “The girl covers the dog and the boy covers the cat.” in which the exhaustive sets of *who* and *which* are pairwise linked.

Children’s experience with such sentences is extremely reduced. Grebenyova (2005, 2011) showed that there are only five instances of such questions in the English CHILDES database. A search through the two corpora on Romanian in CHILDES (MacWhinney, 2000) yielded no instances of multiple *wh-*questions. The acquisition of multiple wh-questions has received relatively little attention in the literature. Grebenyova (2011) elicited multiple interrogatives from 20 monolingual English-speaking children (aged 3;07–6;02), 20 monolingual Russian-speaking children (aged 3;05–6;05) and 18 Malayalam-speaking children (aged 4;05–5;04). The three languages differ with respect to the movement properties of wh-words. Russian allows multiple wh-fronting, while English fronts one wh-phrase and Malayalam is a wh*-in-situ* language. Grebenyova’s findings demonstrate that English- and Malayalam-acquiring children have adult-like knowledge of the syntax of multiple wh-questions, whereas Russian-speaking children allow fronting of only one of the wh-phrases, following an English-like structure.

To our knowledge, three studies so far investigated the acquisition of multiple wh-questions in Romanian and they all looked at how Romanian-speaking monolingual children ranging in age from 4 to 9 years old comprehend this type of question. Bentea (2010) examined how 4- to 6-year-old English, French and Romanian children (24 in total) interpret multiple *wh-*questions (i.e., whether they assign pair-list readings to multiple interrogatives). Bentea (2010) was also interested in whether children assign an adult-like structure to multiple interrogatives in their language and whether cross-linguistic differences appear between English, French and Romanian children regarding the interpretation and structure of multiple questions. Bentea’s (2010) results showed similar performance in the English and French groups, while Romanian-speaking children were more likely to answer only the lower *wh-*element present in the question. In the same vein, Măniță (2017) addressed the question of exhaustivity in the comprehension of Romanian multiple interrogatives. Măniță (2017) tested 42 monolingual Romanian-speaking children (age range 4;0–6;10) and found that the rate with which children give exhaustive answers increases with age, although it does not reach ceiling performance at the age of 6. Furthermore, her results show that children preferentially answered the highest *wh-*word, which was also the subject.

In a recent study on the processing of Romanian multiple *who* and *which*-questions, Bentea and Marinis (2021) show that both monolingual children (6 to 9-year-olds) and adults slow down when processing *who-* compared to *which-*phrases, as measured by reaction times (RTs) in a self-paced listening task. However, only adults seem to show an online sensitivity to the ordering constraints in *who*-questions illustrated in (1b) above. Bentea & Marinis also report higher accuracy scores with multiple *who*- than *which*-questions and show that the latter pose more difficulties for comprehension, particularly in the object-subject order (1,2b), where participants (especially children) show a preference to interpret the first wh-element as agent, along the lines of what has been reported for the processing and comprehension of simple *which-*questions. Bentea & Marinis also found that children even at the age of 6 and 7 answered only one of the wh-phrases, similarly to Bentea (2010) and Măniță (2017), but provided exhaustive lists of referents either for the wh-subject or the wh-object. This suggests that Romanian children have difficulties with pairing the two wh-elements and that this difficulty persists until around the age of 8 when they are able to *exhaust* the question domain and also *pair* the two wh-elements. Therefore, the question that arises is whether bilinguals, who receive less input than monolinguals and are often not tutored in the L1, converge on the correct syntactic structure for multiple wh-questions and attain knowledge of the grammaticality distinctions among *who* and *which*-multiple questions, especially when such wh-dependencies display a different structure in the L2, the societal language.

To sum it up, multiple wh-interrogatives allow to explore the extent to which bilingual children’s language comprehension and production are affected by cross-linguistic influence, as they vary across languages and display language-specific syntactic and semantic properties that children need to acquire although these structures are not frequent in the parental input. In this study, we compare for the first time both the on-line/off-line comprehension and the production of these structures in Romanian heritage children in order to get a clearer picture of the way in which the societal language (here English) influences the acquisition of morphosyntax in the HL.

### Research questions and predictions of the current study

The present study investigates the early stages in the acquisition of the HL to examine whether the differences that emerge between HL children and monolinguals hold not only for production, as has been shown by the previous studies examining the acquisition of simple wh-questions in HL children, but also for comprehension. We postulated that the use of a more sensitive and implicit on-line comprehension task, like the self-paced listening task used in this study, might offer a more straightforward glimpse into underlying language representations that are accessed for real-time processing. Together with production tasks, on-line comprehension might help to better understand what differentiates between HL children and monolingual children. The study focused on Romanian as heritage language and addressed the following research questions:

1. Do Romanian HL children and Romanian monolingual children differ when processing questions with multiple wh-fronting in an on-line processing task?

Previous studies with L2 children looking at real-time sentence processing report qualitatively similar processing patterns in bilinguals and monolinguals for tense (Chondrogianni and Marinis, 2012), articles (Chondrogianni et al., 2015a), articles and clitics (Chondrogianni et al., 2015b), word order (van Dijk et al., 2022). Therefore, we expected Romanian HL children to show similar processing patterns to Romanian monolingual children. On the other hand, if there is cross-linguistic influence from English, the societal language, on Romanian HL children’s processing of multiple wh-questions, regardless of surface overlap (Nicoladis, 2006, 2012), then heritage children should slow down when they hear the second wh-phrase immediately following the first wh-word.

2. Do Romanian HL children and Romanian monolingual children differ with respect to the production of interrogatives with multiple wh-movement and how does this compare to comprehension?

This is the first study to examine the production of multiple wh-interrogatives in Romanian, as previous studies have only looked into how Romanian-speaking children comprehend this type of questions (Bentea, 2010; Măniță, 2017; Bentea and Marinis, 2021). If the Romanian-speaking children tested in Romania have fully acquired the syntax of multiple interrogatives, they should mainly produce questions with multiple wh-fronting. As far as the heritage group is concerned, we base our predictions on the previous studies on the production of wh-dependencies in child HL (Strik and Pérez-Leroux, 2011; Cuza, 2016) which show qualitative differences between HL and monolingual children in the production of wh-questions. We thus expected Romanian heritage children to be more likely to produce multiple wh-questions with one fronted wh-phrase and one *in-situ*, under cross-linguistic from English, the majority language. Moreover, if asymmetries arise between production and comprehension, then we expect the results to show a similar pattern to that reported for other bilingual populations in which comprehension of multiple interrogatives outpaces their use in production (Haiden et al., 2009; Chondrogianni and Marinis, 2012; Chondrogianni et al., 2015a,b).

## Materials and methods

### Participants

Eighteen 6- to 9-year-old Romanian heritage children with English as L2 (6 boys; age range = 6;0–9;3; mean age = 96.6 months; *SD* = 13.7 months) living in the United Kingdom (Greater London area and South-East England) and 30 Romanian monolingual children aged six to nine (15 boys; age range = 6;11–9;8; mean age = 99.1; *SD* = 11.2) living in Romania, participated in the study^1^. None of the monolingual children had a history of speech and/or language delay or impairment, while one bilingual child had mild expressive language delay diagnosed at the age of three and for which she underwent Speech and Language Therapy until the age of six. As this participant’s results at the time of testing did not differ from those of other children, they were included in all subsequent analyses.

Details regarding the bilingual children’s language history, including information about their current use of and exposure to both Romanian and English, were collected using a modified version of the Questionnaire for Parents of Bilingual Children (PABIQ; Tuller, 2015). Three parents did not complete the questionnaire. The language background data obtained show that all children were exposed to Romanian from birth, but had a different age of onset (AoO) of English: one child was a simultaneous bilingual, nine children were exposed to English before the age of two (between 5 and 18 months), and five children were exposed to English after the age of three (between 3 and 5 years). The mean age of onset (AoO) of English was 1;10 years (*SD* = 18 months, range = 0–5;0 years) and the mean length of exposure (LoE) to English was 6;0 years (SD = 21 months, range = 2;1–8;0 years). The language background data for the heritage children illustrated in Table 1. Also show that Romanian is used more at home than English, as determined by a paired *t-*test [*t*(14) = 3.61*, p* = 0.002], whereas English is the dominant language outside the home [*t*(14) = 3.62*, p* = 0.003], as well as when it comes to children’s current expressive language skills, as reported by the parents [*t*(14) = 4.45*, p* < 0.001].

**Table 1:** Language background information for the heritage Romanian group.

		Romanian	English
Amount of exposure before 4yo	*M (SD)* *MIN-MAX*	0.82 (0.20) 0.50–1.00	0.45 (0.29) 0–1.00
Parental ratings of current skills	*M (SD)* *MIN-MAX*	0.51 (0.23) 0.20–1.00	0.85 (0.18) 0.40–1.00
Language use at home	*M (SD)* *MIN-MAX*	0.64 (0.23) 0.17–1.00	0.40 (0.18) 0.20–1.00
Current exposure outside the home	*M (SD)* *MIN-MAX*	0.44 (0.18) 0.14–0.71	0.70 (0.19) 0.36–1.00

### Tasks

#### Self-paced listening task

Children’s ability to comprehend Romanian multiple wh-questions in real-time was assessed with an on-line SPL task with picture verification [see Marinis and Saddy (2013)]. In this task, participants reaction times (RTs) are measured every time they press a key on the keyboard in order to listen to sentences presented word-by-word or phrase-by-phrase. The advantage of using such a task is that children administer the auditory stimuli at their own pace and this gives an indication of how fast they process each word/phrase in the sentence.

The self-paced listening task in the current study was presented as a computer game with a puppet (Paddington the Bear). The children were told that they have to listen very carefully to Paddington’s questions in order to be able to identify the correct characters in the picture that followed each question. The experimental items contained embedded questions with two fronted wh*-*phrases in which we crossed two factors: the order of the wh-constituents [either the wh-subject preceded the wh-object (SO) or *vice-versa* (OS)] and the type of wh-phrase (*who* vs. *which*). There were 40 test items in total, with 10 items per condition. The sentences included stories about superheroes (Superman, Batman, and Spiderman) and princesses (Anna, Elsa, and Jasmine) engaging in imaginary activities with different people or animals. At the beginning of every trial, children listened to a lead-in sentence, introducing the characters. This was then followed by the test sentence (i.e., an embedded question) segmented into 8 parts, each part containing one to three words. No images appeared on the screen while children listened to the lead-in and the test sentence. The slashes in the examples signal the end of each segment when children had to press the Space bar to hear the next segment. After the final segment of each test sentence, a picture with three pairs of characters appeared on the screen. All the visual stimuli used in the study can be provided upon request. The position of the pairs and the direction of the actions varied between pictures. Children had to verbally identify all the pairs of characters performing the action described in the sentence. An answer was coded as “correct” when all the relevant pairs were identified. After hearing the sentence in (3), for example, the expected answer was *The fireman is splashing Superman and the elephant is splashing Batman.* This is because Romanian only allows exhaustive pair-list interpretations for multiple wh-questions like the ones exemplified in (3) to (6) below.

Lead-in:    This is an image of Spiderman, Superman, Batman, a boy, a fireman and an elephant.
  Test sentence: Paddington/ wants to know/
   3. *SO who*  **cine**/**pe cine**/ stropește/în joacă/seara/           la circ/
          who PE whos plashes jokingly in the evening        at the circus
          “who is splashing whom jokingly at the circus in the evening.”
   4. *OS who*  *pe cine/ **cine**/ stropește/în joacă/  seara/          la circ/
          PE who   who splashes  jokingly    in the evening       at the circus
          “who whom splashing jokingly at the circus in the evening.”

Lead-in    This is an image of Anna, Elsa, Jasmine, and three monkeys.
  Test sentence: Paddington/ wants to know/
   5. *SO which*  **care  prințesă**/**pe care  maimuță**/  o/  acoperă/  dimineața/      la zoo/
          which princess  PE which monkey      her   covers    in the morning    at the zoo
          “which princess is covering which monkey at the zoo in the morning.”
   6. *OS which*  **pe care prințesă**/**care    maimuță**/  o/  acoperă/  dimineața/      la zoo/
          PE which princesswhich monkey        her  covers     in the morning    at the zoo
          “which princess is which monkey covering at the zoo in the morning.”

Apart from the 40 test items, 10 fillers were also included as distractors. Half of the fillers were simple subject *who*-questions and the other half were simple subject *which*-questions. They included both transitive actions (*eat, hold, read, cut, smell*) in which the agent was always animate and the patient inanimate, as well as intransitive actions (*fly, jump, run, sleep*).

#### Elicited production task

For the elicited production task, children played a guessing game with Paddington the Bear in which they were prompted to produce 24 multiple wh-questions with a subject-object and an object-subject order. Six items were designed to elicit multiple *who-*questions with a SO order (7), 12 items elicited multiple *which-*questions (six with a SO order as in (8) and six with an OS order as in (9)), while six more items were designed to elicit multiple wh-questions with an OS order (10), in which the object was a *which*-phrase and the subject a *who*-phrase, a grammatical option in Romanian.

7.*SO who*     **Cine  pe   cine**  a  mângâiat?
            who   PE   who  has patted
            “Who patted whom?”
  8.*SO which*     **Care  fată  pe   care  pisică**  a   mângâiat-o?
            which  girl  PE   which  cat    has  patted-her
            “Which girl patted which cat?”
  9.*OS which*     **Pe  care   pisică  care  fată**   a    mângâiat-o?
            PE  which   cat    which  girl   has   patted-her
            “Which cat did which girl pat?”
  10.*OS which-who*  **Pe  care  pisică  cine**   a    mângâiat-o?
            PE   which  cat     who   has   patted-her
            “Who patted which cat?”

Children interacted with a Paddington the Bear puppet for the whole duration of the task (see details in the Procedure section below).

The structure of each scenario was as follows. First, both the child and Paddington saw an image of Lego figures and heard *Here are two girls, a boy, two cats and a monkey*, which helped them to familiarise themselves with the characters in the image. The experimenter then covered Paddington’s eyes and ears so that the puppet could not see or hear anymore what was happening next in the image. At this point, the child saw another image and heard *Look! This girl is patting the black cat and this girl is patting the white cat. The boy is taking a picture of the monkey.* Then the image presented at the beginning of the trial appeared again on the screen. Paddington’s eyes and ears were uncovered and afterwards the experimenter would tell the puppet *Paddington, we can tell you that the boy did not pat anyone, but each girl patted a different cat.* The experimenter then prompted the child to ask Paddington a question about this (e.g., *Which girl patted which cat?*) and then Paddington made his guess. In order to make sure that children produced questions with two wh-phrases, they were told that they need to ask questions about two things at the same time. The task also included six filler items. These prompted children to produce simple wh-questions (three argument questions with a mismatch in animacy like *“What did the father wash?* and three adjunct questions such as *Where did the queen sit?*). Children’s responses were coded as “felicitous” when they produced a target question with multiple wh-movement like in examples (7) to (10) above. Other responses were coded as “infelicitous,” alongside the type of error produced.

### Procedure

The study was approved by the Ethics Committee of the University of Reading. Informed parental consent was obtained for each child prior to the testing sessions, as well as the oral consent of the child. Each participant was tested individually in a quiet room either at their school or in their home. The experimenter gave oral instructions for both tasks. These were administered at least 1 week apart and in different orders, such that half of the children first saw the SPL task, followed by the elicited production task, while for the other half we first assessed production and then comprehension. Each testing session lasted around 30–40 min, but children could take breaks whenever they felt tired. They received stickers and certificates of participation after each task and at the end of the study they received as well a voucher which they could use to buy books at local bookstores.

The SPL task was programmed and administered using PsychoPy (Peirce et al., 2019). The task started with an introduction in which an image of Paddington first appeared on the screen, telling children what they need to do in the task. Children were also familiarized with the images and names of each of the three superheroes and princesses, although almost all them already knew these characters from cartoon movies. Before starting the test phase, the children were presented with four practice items, two of which contained simple *what*-questions (e.g., *What* is Batman reading?) and two multiple wh-questions with an animate subject and an inanimate object (e.g., *Who* is drinking *what*?). Each participant then listened to a total of 50 sentences during the test phase and these were randomized in PsychoPy. The task instructions and all the sentences were pre-recorded by a native speaker of Romanian.

The elicited production task was administered as a PowerPoint presentation. The task started by introducing each child to the Paddington puppet. The experimenter explained that Paddington wanted to become a magician and for this he had to improve his guessing skills. The child’s task was to help Paddington by playing a game with him in which the child asked Paddington questions about various images and the puppet had to guess the correct answer. For each correct guess, the child gave Paddington a smiley face sticker and if Paddington had at least 20 correct guesses by the end of the task, the child gave Paddington his Magician Diploma. Given the complexity of the target questions, these were presented in four blocks and interspersed with fillers. Each block contained 6 items of the same type (e.g., *SO who* questions) and the order of the blocks was randomized across four lists, each containing 30 items. The task began with four practice trials which elicited multiple wh-questions with a mismatch in animacy (e.g., *Who ate what?* or *Which boy hid where?*). The audio presentation of the items was also pre-recorded by a native speaker of Romanian. The questions that the children produced were recorded on answer sheets and then coded for analysis.

## Results

### Comprehension of multiple wh-dependencies

**Figure 1:**
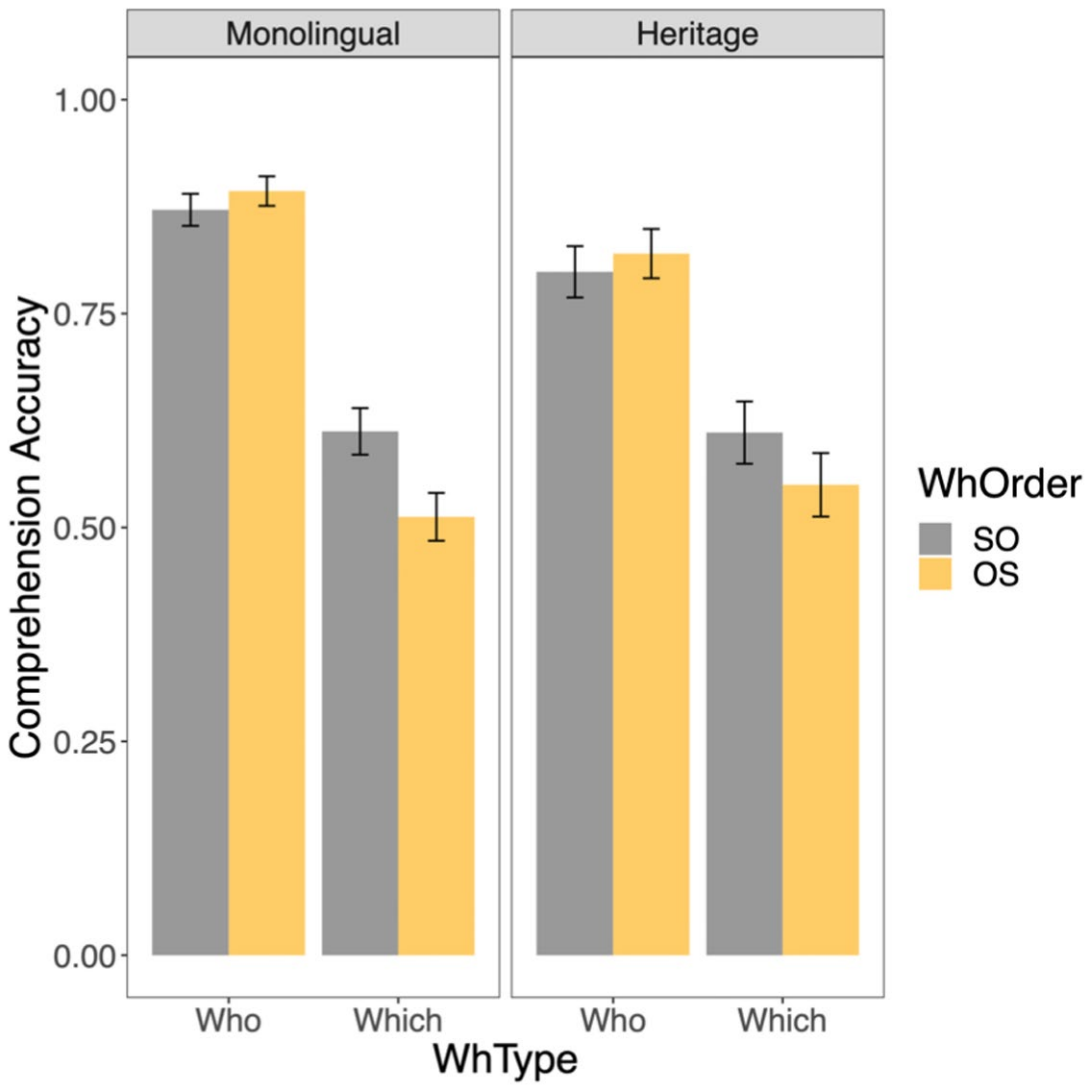
Mean accuracy scores (with standard error bars) for multiple wh-questions per group (Monolingual vs. Heritage) per wh-type (*who* vs. *which*) per wh-order (SO vs. OS).

Figure 1 presents the descriptive results for **comprehension accuracy** of Romanian multiple wh-questions with two *who* and two *which-*phrases and with distinct subject-object orders. The results indicate that both the Romanian-speaking monolinguals and the heritage Romanian children comprehend multiple *who-*questions well (above 0.80), but show lower accuracy for multiple *which-*questions.

The two groups also show similarities in the errors they produce, which are (i) over-exhaustive answers, (ii) list answers, and (iii) reversed role answers. For over-exhaustive answers children name all the pairs in the image, even when one pair performs a different action. For example, children would answer a question like in (3) or (4) above with *The fireman is splashing Superman, the elephant is splashing Batman, and the boy is pulling Spiderman* although the question only refers to the action of splashing*.* List answers are cases in which children only answer one of the two wh-words by exhaustively listing all the characters involved in the action. That is, they would answer question (5) with *Jasmine and Elsa*. Role reversals are answers in which the Agent-Patient roles are reversed. For instance, children would answer the question in (6) with *Anna is covering the black monkey* and thus assign the wrong thematic role to *which princess*. There were also very few instances in which children simply identified the wrong action, for example the pulling action when the question was about splashing. Table 2 summarizes the number and type of errors for monolingual and heritage children and indicates that the most common errors children make with multiple *who-*questions are providing list answers to only one of the wh-words, while role reversal errors are the most frequent with multiple *which*-questions. This suggests that children find it harder to assign the correct thematic roles in a patient-before-agent structure when the question contains two *which-*phrases.

**Table 2:** Type and number of errors by group for comprehension accuracy of multiple wh-questions (total number of errors per condition in parantheses).

Group	Condition	Error types			
		Over-exhaustive	List answers	Reversed role	Wrong action
Monolingual	Who	16 (*76*)	56 (*76*)	0 (*76*)	4 (*76*)
	Which	32 (*280*)	63 (*280*)	185 (*280*)	0 (*280*)
Heritage	Who	6 (*69*)	59 (*69*)	0 (*69*)	4 (*69*)
	Which	25 (*151*)	53 (*151*)	75 (*151*)	0 (*151*)

Given the binary nature of the data (Correct/Incorrect), we analyzed the accuracy results using a binomial generalized linear mixed model with group (monolingual vs. heritage), wh-type (who vs. which), wh-order (SO vs. OS), and their interaction as fixed factors. The fixed factors were coded using repeated contrast coding which tests the difference between the mean of the dependent variable for one level of the categorical variable and the mean of the dependent variable for the adjacent level (Schad et al., 2020). The random-effects structure included intercepts for both participants and items, as well as random slopes for wh-type by participant. Alternative models with a more complex random-effect structure either failed to converge or were not retained as their goodness-of-fit resulted in increased Akaike information criterion (AIC)-value. The analysis was conducted using the *lme4* package (Bates et al., 2015) in R (R Core Team, 2022). Planned comparisons, if justified, were done using the *emmeans* package (Lenth, 2022).

The three-way interaction group*wh-type*wh-order did not significantly improve the fit of the model as indicated by model comparison using the anova function (*p* = 0.988). The summary of the final model is given in Table 2. The results showed a significant effect of wh-type (*who*-questions were comprehended better than *which*-questions) and a significant wh-type*wh-order interaction. No differences in performance emerged between the monolingual and the heritage group (Table 3).

**Table 3:** Model summary for comprehension accuracy of multiple wh-questions.

	Estimate	SE	*Z*	Sig.
(Intercept)	1.78317	0.32066	5.561	**<0.001**
group(monolingual vs. heritage)	−0.08496	0.60404	−0.141	0.888
wh-type(who vs. which)	2.89120	0.39958	7.236	**<0.001**
wh-order(SO vs. OS)	0.00179	0.15963	0.011	0.991
group_monolingual vs. heritage_* wh-type_who vs. which_	0.06594	0.68398	0.096	0.923
group_monolingual vs. heritage_* wh-order_SO vs. OS_	0.12909	0.26980	0.478	0.632
wh-type_who vs. which_* wh-order_SO vs. OS_	0.80690	0.30057	−2.685	**0.007**
Observations	1995 0.220/0.663			
Marginal *R*^2^/Conditional *R*^2^				

The values in bold indicate statistical significance.

We followed-up on the significant interaction between wh-type and wh-order with a pair-wise comparison. This showed that response accuracy for questions with two *who-*phrases did not differ significantly for the SO and OS orders (*β* = −0.402, *SE* = 0.271, *z* = −1.480, *p* = 0.138), while there was a significant difference between the two wh-orders in the case of questions with two *which*-words, with SO *which* questions yielding significantly better comprehension scores than OS *which* questions (*β* = 0.405, *SE* = 0.150, *z* = 2.703, *p* < 0.01).

**Figure 2:**
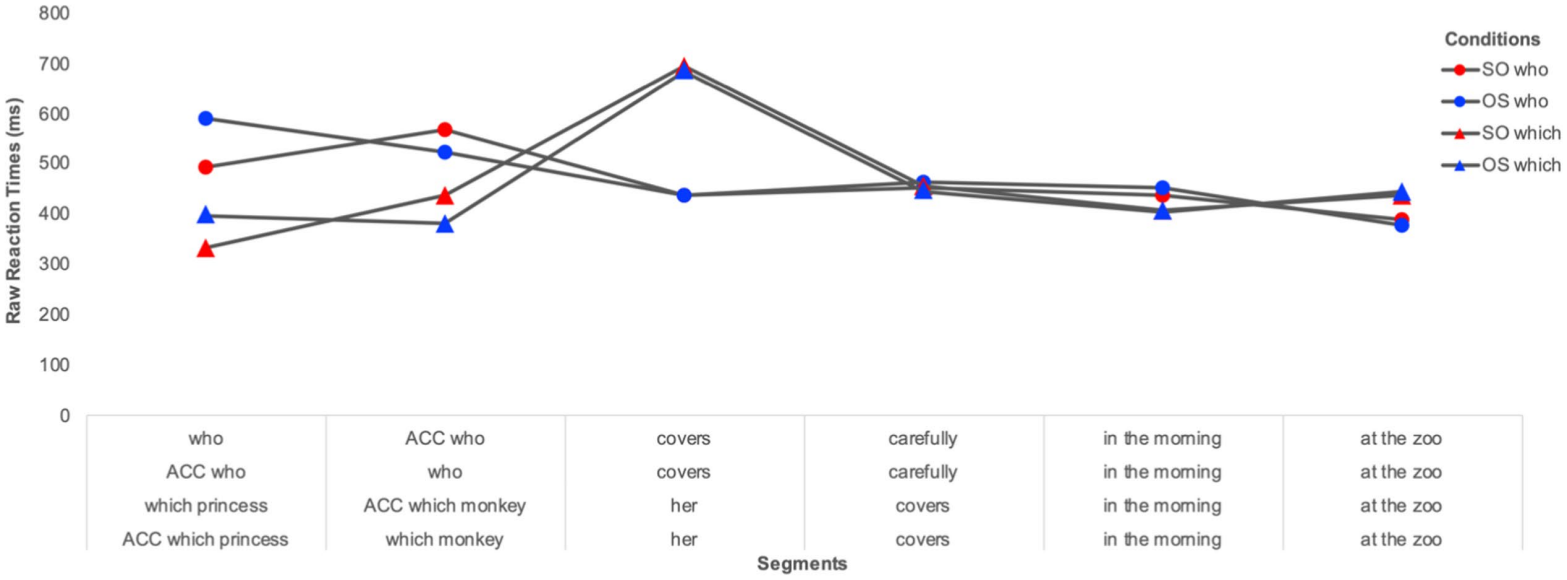
Segment-by-segment distribution of residual RTs per wh-type (*who* vs. *which*) and per wh-order (SO vs. OS) for Romanian-speaking monolingual children.

**Figure 3:**
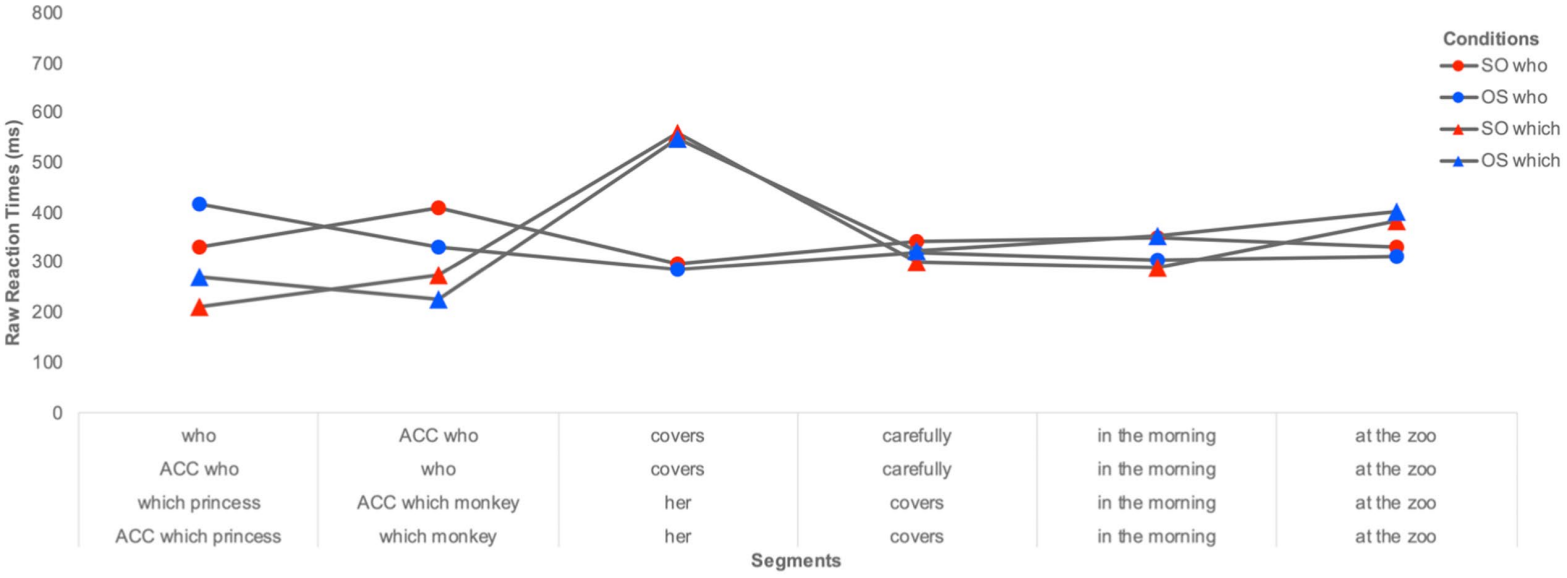
Segment-by-segment distribution of residual RTs per wh-type (*who* vs. *which*) and per wh-order (SO vs. OS) for heritage Romanian children.

The segment-by-segment residual reaction times (RTs) for the **online processing** of multiple wh-questions are illustrated in Figure 2 (for the monolingual group) and Figure 3 (for the heritage group). We plot raw residual RTs for readability, but the analyses were done on log-transformed RTs. Only items with correct responses to the comprehension questions were included in the RT analyses. Residual RTs were calculated to control for the difference in length between segments. Extreme values were calculated based on boxplots and were excluded from the final analyses. These were residual RTs below −600 ms and above 2000 ms. RTs of 2 *SD* above or below the mean per condition per participant and per item were considered as outliers and therefore replaced with the mean per condition per participant and per item. The total proportion of extreme values and outliers was 3.2% of all data points.

We report the results from six segments, starting with the segment containing the first wh-word and including the segments after the verb, as these can reveal spill-over effects. The log-transformed RTs for each segment were analysed using linear mixed-effects models fit with the maximal random effects structure that converged. This included by-participant and by-item random intercepts and slopes for wh-type by-participant. Group (Monolingual vs. Heritage), wh-type (who vs. which), wh-order (SO vs. OS), as well as their interaction, were included in the fixed effects structure for each model. All fixed effects were coded using repeated contrast coding. Values of *p* were calculated by way of Satterthwaites’s approximation to degrees of freedom with the *lmerTest* package (Kuznetsova et al., 2017).

*Segment 1 (the first wh-word)* Results attested to a significant effect of group (β = 0.178, SE = 0.057, *t* = 3.121, *p* = 0.003), indicating that the monolingual children had overall longer RTs than the heritage children. There was also a significant effect of wh-type (β = 0.237, SE = 0.042, *t* = 5.522, *p* < 0.003), with longer RTs for *who*-phrases (M = 482 ms) than *which*-phrases (317 ms), as well as a significant effect of wh-order (β = −0.178, SE = 0.057, *t* = 3.121, *p* = 0.003), with shorter RTs for subject (M = 371 ms) compared to object wh-words (458 ms). None of the interactions was significant.

*Segment 2 (the second wh-word)* Results revealed a significant effect of group (β = 0.143, SE = 0.055, *t* = 2.564, *p* = 0.013), with the monolingual children displaying longer RTs than the heritage children, and a significant effect of wh-type (β = 0.167, SE = 0.034, *t* = 4.845, *p* < 0.001), with longer RTs for *who*-phrases (M = 484 ms) than *which*-phrases (352 ms). No other effect or interaction were significant.

*Segment 3 (the verb in multiple who-questions and the clitic in multiple which-questions)* There was a significant effect of group (β = 0.190, SE = 0.084, *t* = 2.266, *p* = 0.028), with longer RTs for the monolingual group compared to the bilingual group, and a significant effect of wh-type (β = −0.436, SE = 0.046, *t* = −9.291, *p* < 0.001) showing that the verb segment in the multiple *who*-conditions yielded shorter RTs (M = 386 ms) than the clitic segment in multiple *which*-conditions (M = 638). There was also a significant interaction Group*WhType (β = 0.151, SE = 0.051, *t* = 2.910, *p* = 0.003). As a follow-up on the significant interaction, pair-wise comparisons revealed that the heritage group showed significantly shorter RTs than the monolingual group for the *who*-conditions, (β = −0.266, SE = 0.086, *t* = −3.065, *p* = 0.003), while no significant differences surfaced between the two groups in the *which*-conditions.

*Segment 4 (the adverb in multiple who-questions and the verb in multiple which-questions)* No effect was significant.

*Segment 5 (in the morning)* Results attested to a significant effect of group (β = 0.149, SE = 0.072, *t* = 2.057, *p* = 0.045), as the monolingual children had longer RTs than the heritage children. No other effect was significant.

*Segment 6 (at the zoo)* No effect was significant.

To summarize, the results of the comprehension task show similar response accuracy and a similar pattern during online comprehension of multiple wh-questions in Romanian-speaking monolingual and heritage Romanian children.

### Production of multiple wh-dependencies

The results of the elicitation task showed that children do not only produce questions with multiple wh-fronting, the expected target structure based on the syntax of Romanian multiple wh-questions, but that they often produce other structures as well. To reflect this variability in the children’s answers, four scoring categories were used, each corresponding to the four main question types that children produced and which we classified as follows:

a. MWH_MULTIPLEMOVE: when children produced a question with two wh-words, either *who* or *which*, and with both wh-words fronted, as in (11). 
  11. **Cine pe cine**   a mângâiat ?
      who PE whom has patted
      “Who patted whom?”
b. MWH_SINGLEMOVE: when children produced a question containing two wh-words in which only one wh-phrase is fronted and the other one appears in-situ, like in (12). 
  12. **Cine** a mângâiat **pe cine** ?
      who has patted PE whom
      “Who patted whom?”
c. SIMPLE_WH: when participants produced a grammatically correct question but only with one fronted wh-word (13). 
  13. **Care  fată** a mângâiat pisica?
      which girl  has patted  cat.the._F.SG_
      “Which girl patted the cat?”
d. COORDINATED_WH: when children produced a question with two coordinated wh-words (14). 
  14. **Cine și  pe cine** a mângâiat ?
      who and PE who has patted?
      “Who and whom is punching?”

**Figure 4:**
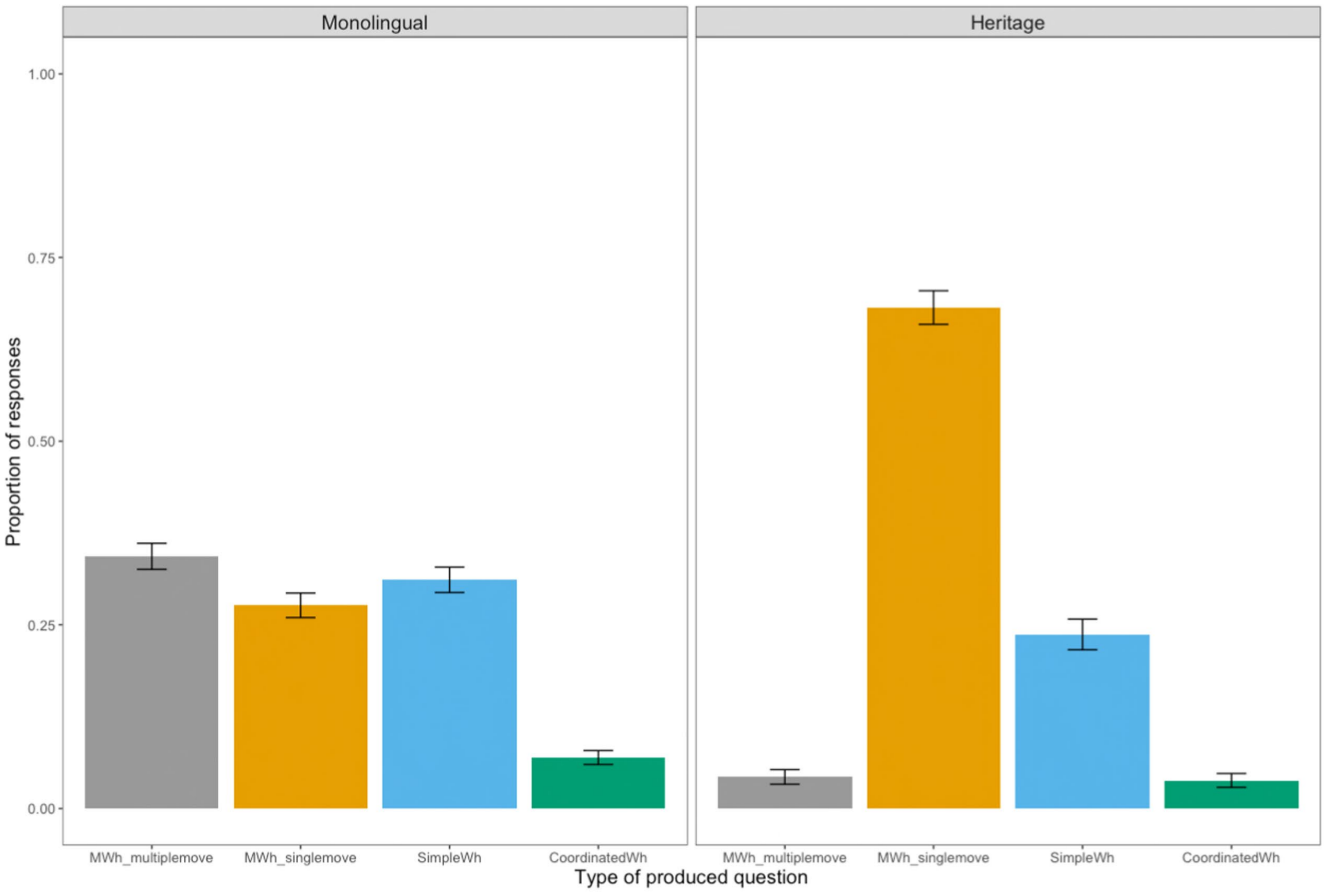
Overall distribution of responses per Group (Monolingual vs. Heritage).

The distribution of responses differs between the Romanian monolingual and Romanian heritage children, as can been seen in Figure 4. Monolingual children produce three types of questions at similar rates: MWH_MULTIPLEMOVE (0.34), MWH_SINGLEMOVE (0.28) and SIMPLE_WH (0.31). They also produce COORDINATED_WH to a lesser extent (0.07). The most frequent type question that the heritage children produce is MWH_SINGLEMOVE (0.68), followed by SIMPLE_WH (0.24). There are also a few instances of COORDINATED_WH questions (0.04), as well as instances of MWH_MULTIPLEMOVE questions (0.04). However, a closer look at the data reveals that the majority of multiple wh-questions with two fronted wh-phrases are produced by one child and that there are six other children who only produce one question with multiple wh-movement throughout the whole task.

**Figure 5:**
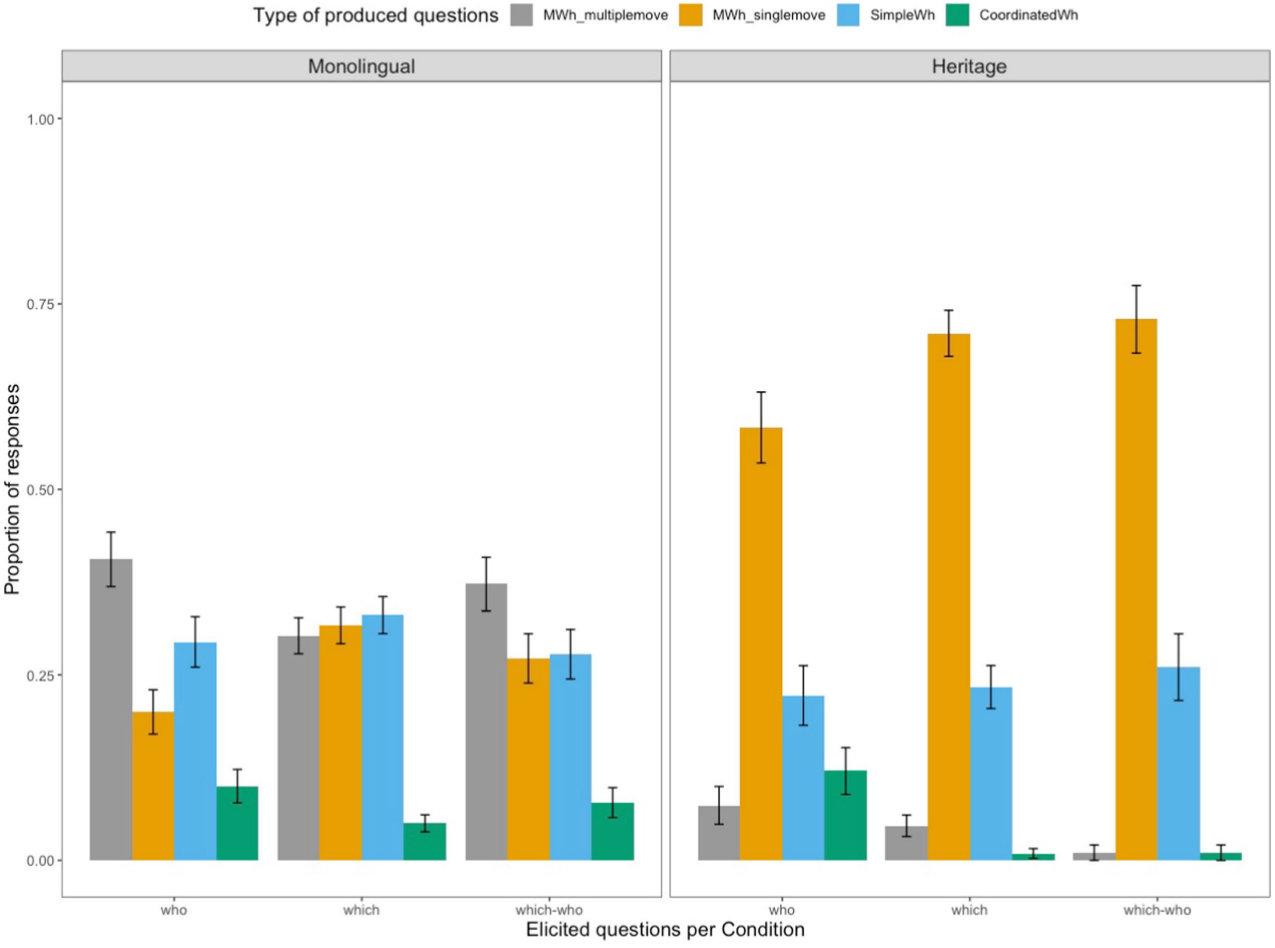
Mean production proportion (with standard error bars) for each type of produced question per Group (Monolingual vs. Heritage) and per Question Type (who, which, which-who).

A finer characterization of the results (Figure 5) gives an indication of the response distribution within the two groups of participants for each type of elicited question (see examples 7 to 10 above). As the children mainly produced questions with a subject-object order, we collapsed the results of *SO which* and *OS which* questions and thus report the data for three types of multiple wh-questions with two *who*-phrases (who), with two *which-*phrases (which), and with one *which* and one *who* phrase (which-who). Figure 5 shows that MWH_SINGLEMOVE questions (with one wh-phrase fronted and one *in-situ*) represent the preferred produced structure for the heritage group across all types of elicited questions, irrespective of whether these contained only *who*, only *which*, or both *which* and *who* phrases. The monolingual group produce more MWH_SINGLEMOVE structures of the type illustrated in (12) with questions containing *which-*phrases, whereas they produce more MWH_MULTIPLEMOVE structures like in (10) in the presence of two *who-*elements*.*

In order to uncover whether differences emerge between monolingual and heritage children in their productions and whether these differences are modulated by the type of elicited question (*who* vs. *which* vs. *which-who*), we fitted a generalized liner mixed model for each of the four scoring categories outlined above, namely MWH_MULTIPLEMOVE, MWH_SINGLEMOVE, SIMPLE_WH, COORDINATED_WH. The dependent variable in each model was response accuracy, that is, the correct production of questions within each scoring category. We analyzed the production data using the *lme4* package (Bates et al., 2015) in the R environment (R Core Team, 2022), specifying the optimizer ‘bobyqa’ for our models. Each model included the same fixed factors (Group: Monolingual vs. Heritage and QuestionType: *who* vs. *which* vs. *which-who*) as well as their interaction. We used a repeated contrast specification for the fixed factors. The random effect structure included by-participant random slopes. We only report the significant effects and interactions.

#### MWH_MULTIPLEMOVE

The statistical analysis revealed a main effect of Group (β = −4.659, SE = 1.102, *z* = −4.225, *p* < 0.001). Overall, the heritage children produced significantly fewer questions with multiple wh-fronting compared to the monolingual children. The analysis also revealed a significant difference between *which* and *who* questions (β = −0.957, SE = 0.362, *z* = −2.643, *p* = 0.008). Multiple wh-fronting was most frequent when children had to produce questions with two *who*-phrases compared to two *which-*phrases. The interaction Group_Heritage *vs* Monolingual_*QuestionType_which-who *vs* which_ was also significant (β = −2.904, SE = 1.220, *z* = −2.381, *p* = 0.017). As a follow-up on the significant interaction, pair-wise comparisons with an adjusted alpha level using the Tukey method showed that the monolingual group produced significantly fewer questions with multiple wh-movement when the target items only contained *which*-phrases compared to when they contained one *who*-phrase and one *which*-phrase (*which – which-who:* β = −0.726, SE = 0.299, *z* = −2.432, *p* = 0.039).

#### MWH_SINGLEMOVE

The statistical analysis revealed a main effect of Group (β = 3.667, SE = 1.123, *z* = 3.265, *p* = 0.001). This means that, overall, heritage children produced significantly more multiple wh-questions with one element fronted and one *in-situ* than the monolingual children. There was also a significant difference between *which* and *who* questions (β = 1.849, SE = 0.305, *z* = 6.054, *p* < 0.001), indicating that there were overall more multiple wh-questions with single wh-fronting for *which*-questions relative to *who*-questions.

#### SIMPLE_WH

There was no difference between the two groups on this measure.

#### COORDINATED_WH

The statistical analysis revealed a significant difference between *which* and *who* questions (β = −2.994, SE = 0.580, *z* = −5.155, *p* < 0.001). This means that children produced less coordinated wh-questions when both wh-words elements were *which*-phrases. There was also a significant Group_Heritage *vs* Monolingual_*QuestionType_which *vs* who_ interaction (β = −2.479, SE = 1.157, *z* = −2.141, *p* = 0.032). Subsequent pairwise comparisons show that the heritage group produces significantly fewer coordinated wh-questions when the target items contained two *which-*phrases compared to when these contained two *who-*phrases (β = −4.234, SE = 1.011, *z* = −3.091, *p* < 0.001) and this difference is more pronounced in heritage children than in monolinguals.

Thus, heritage children display a different pattern than monolinguals in production, as they produce significantly fewer questions with multiple wh-fronting and significantly more questions with one fronted wh-phrase and one *in-situ* compared to the monolingual children.

## Discussion

The main questions guiding the present study were whether differences emerge between Romanian-English bilingual children, for whom Romanian is the heritage language, and Romanian monolingual children in the on-line comprehension and production of multiple wh-interrogatives and whether these differences are due to cross-linguistic influence from the dominant English. To address these questions, we used an on-line comprehension task and an elicitation task targeting multiple *who-* and *which-*questions. The on-line task investigated how Romanian heritage children and Romanian monolingual children process wh-dependencies with two fronted wh-words, while they were listening to sentences on-line for comprehension. This task also aimed to find out whether Romanian-speaking children were sensitive to the asymmetry in object-over-subject movement between multiple *who*- and *which*-questions in real-time comprehension. The combination of production and on-line comprehension tasks can help to get a better picture of heritage language development and better understand the relationship between performance in production and real-time processing.

The results of the comprehension task reveal similar accuracy for the interpretation of multiple wh-questions in Romanian heritage and monolingual children. Both groups comprehend questions with a fronted *who*-subject and *who*-object well, irrespective of the order in which the two wh-phrases occur. In contrast, they have more difficulties with the comprehension of multiple *which-*question, particularly when the wh-object precedes the wh-subject. This is in line with cross-linguistic findings reported for the comprehension of simple *which-*questions showing that children find object *which-*questions harder to comprehend than subject *which-*questions and that the type of wh-element also affects comprehension of wh-dependencies, with object *who-*questions being acquired earlier thn object *which-*questions (Friedmann et al., 2009; Bentea and Durrleman, 2013; Contemori et al., 2018).

The self-paced listening data also show a similar pattern during on-line comprehension of multiple interrogatives in Romanian heritage and monolingual children, with shorter RTs when processing *which* vs. *who-*phrases. This finding, coupled with the offline response results, reflects a speed-accuracy trade-off: children are more accurate with *who*- than *which-* multiple interrogatives, but they slow down when they process *who-* as compared to *which-*phrases. This difference in processing between wh-elements has also been attested for English by Hofmeister et al. (2013) who tested English-speaking adults in a self-paced reading task and reported more efficient processing in English multiple wh-questions for *which*-constituents compared to *who-*phrases. Moreover, Romanian heritage-children do not slowdown upon listening to the second wh-phrase, as we predicted would be the case if their processing of multiple wh-dependencies in Romanian would be affected by cross-linguistic influence from the dominant English. Furthermore, neither group showed an on-line sensitivity to the ungrammatical object-subject order in multiple *who-*questions, contrary to the findings for Romanian monolingual adults in Bentea and Marinis (2021). The authors show that, adults, but not children, are sensitive to the ungrammaticality of multiple *who*-questions in which the wh-object precedes the wh-subject and one explanation they put forth is that this effect is delayed in children, in other words that it might only surface after the end of the sentence. Other visual world studies have also found young children to be slow in processing wh-dependencies (Contemori et al., 2018), with effects occurring after the end of the sentence (Adani and Fritzsche, 2015). Structures like multiple wh-interrogatives are more complex than simple wh-questions, as they require encoding, integrating, and retrieving two wh-elements in the structure. This added complexity may delay processing in children even more.

Taken together, the results of the on-line comprehension task corroborate previous findings that looked at real-time sentence processing in L2 children and found qualitatively similar processing patterns in bilinguals and monolinguals (Chondrogianni and Marinis, 2012; Chondrogianni et al., 2015a,b). Our findings also show that, at the quantitative level, Romanian heritage children process sentences at a faster rate than monolingual children (see van Dijk et al. (2022) for similar results with Dutch bilingual and monolingual children). The faster processing behavior observed in the bilingual group in the self-paced listening task could potentially suggest a general effect of bilingualism on sentence processing in children, which could result in more efficient sentence processing. However, more research is needed to explore this observation further. The key finding remains that heritage children do not differ qualitatively from monolingual children and display a similar on-line comprehension pattern to monolingual children for multiple questions in Romanian. When they encounter multiple fronted *who-* and *which*-questions they are able to parse them incrementally in the same way as monolingual children of the same age. They process *which* wh-words faster than the *who* wh-words at the beginning of the sentence and subject wh-phrases faster than object wh-constituents. This suggests that the processing of some syntactic dependencies is preserved in child HL. More studies are however needed to confirm the possible absence of cross-linguistic influence on processing strategies in the HL, as well as to uncover the role that language dominance plays in child HL processing. Our findings seem to be at odds with those of Van Dijk et al. (2022) who report effects of cross-linguistic influence on the processing of V2 structures in Dutch by German-Dutch bilingual children. These effects were more pronounced the more dominant the children were in German. Van Dijk et al.’s results also show that such CLI effects were stronger in instances of partial structural overlap between German and Dutch and were evident as inhibition during listening. In other words, the German-dominant children slowed down when listening to structures in Dutch that had a similar V2 order in German. Although the Romanian-English bilingual children in this study were dominant in English, as measured through their current expressive skills reported by the parents, we did not find evidence of cross-linguistic influence on the processing of multiple interrogatives in Romanian. One potential explanation is the lack of overlap in surface structure between multiple interrogatives in Romanian and English. Future research should thus address the role that structural overlap and language dominance have in modulating online processing in the heritage language.

Let us now turn to the elicitation task which examined whether Romanian heritage and monolingual children are able to produce questions with multiple wh-movement and whether they have fully acquired the specific syntax of multiple interrogatives in Romanian, which requires fronting of all wh-phrases. Contrary to comprehension, we found differences in the production of multiple wh-questions between Romanian heritage and monolingual children. Monolingual children produce questions with multiple wh-fronting (mainly SO *who*), but also questions in which only one wh-phrase is fronted, the other one remaining *in-situ*. This option exploited in production surfaces mostly in questions with two *which-*phrases. In addition, monolingual children also produce a significant number of simple wh-questions, that is, questions with only one wh-word. While heritage children also produce simple questions, they produce significantly more questions with one fronted wh-phrase, one *in-situ* than monolingual children and, with the exception of one child, avoid multiple wh-movement, contrary to monolinguals. Importantly, there were no instances of multiple *who*-questions with an ungrammatical object-subject order in any of the children’s productions. This indicates that their grammatical system does not allow this option and further reinforces the idea that their lack of sensitivity to the object-over-subject ungrammaticality in multiple *who-*questions in on-line comprehension is not due to a different grammar than that of adults, but it most likely stems from the processing load associated with such complex structures involving multiple movement dependencies.

The results for production thus suggest that heritage children seem to opt for a less complex structure that involves fronting of only one wh-phrase. Monolingual Romanian children also make use of a range of structures when prompted to produce wh-questions with multiple fronting, pointing to the fact that they avoid as well the complexity associated with multiple wh-interrogatives which require movement of two wh-phrases. Importantly, what the results of the monolingual children show is that they also employ two structural options to derive multiple interrogatives, the multiple wh-fronting and the one wh-moved, one wh-*in situ* option. Given that the predominant response pattern for multiple wh-questions in Romanian heritage children makes use of the only structural option present in English, we take this to show that there is cross-linguistic influence from the dominant societal language to the heritage language. Similarly, other studies have linked the differences in performance between child heritage speakers and monolinguals to the properties of the societal language (Meir et al., 2017; Meir and Janssen, 2021). The use of the one wh-fronted, one *in-situ* option is reinforced in Romanian under influence from English, the dominant language for the heritage children (Hulk and Müller, 2000; Serratrice, 2013). Our findings for production also match those reported by Strik and Pérez-Leroux (2011) for the production of wh-questions in Dutch by Dutch-French bilingual children, who were French dominant, and who also produced instances of wh *in-situ* in Dutch, a less complex option than wh-fronting. The results from the current study, together with those of Strik and Pérez-Leroux (2011) seem to suggest that structures which require less complex structural derivations (such as one wh-fronted, one *in-situ*) are acquired earlier and also that they are more likely to influence structures which require more complex derivations (like multiple wh-fronting), in line with a complexity-based theory of transfer (Strik and Pérez-Leroux, 2011).

The question that remains is why cross-linguistic influence occurred in children’s production but not in their comprehension. When comparing the results for both comprehension and production of multiple wh-dependencies in Romanian we observe that heritage children are able to establish the underlying representation of multiple wh-movement structures, similarly to monolinguals when they encounter multiple fronted wh-movement structures, but have difficulties activating the more complex structure in production. Such comprehension/production asymmetries have been attested in the majority language of bilingual children for tense (Chondrogianni and Marinis, 2012), articles (Chondrogianni et al., 2015a,b), articles and clitics (Chondrogianni et al., 2015a,b) and have been taken as evidence in favour of the claim that underlying syntactic representations are intact in child L2 acquisition even if non-target-like structures appear in production (Haznedar and Schwartz, 1997). This suggests that in comprehension, where the referents and the linguistic structure are given, children can parse and assign an interpretation to the structure. In doing so, they have to keep in working memory information about the wh-fronted elements and then retrieve them in order establish the correct dependencies between the moved wh-phrases and the verb. In production, on the other hand, they have to start at the conceptual level, they have to plan and build the structure themselves by deciding about the thematic role, case, grammatical function, and syntactic position of the wh-phrases as they speak. (Momma, 2021). In other words, comprehension requires children to recognize the meaning of words and the syntactic dependencies in which the words enter. But in the light of production, children must actively plan the structure and its complexity impacts on this. It is thus more economical to start from a simpler structure than generating a more complex structure, particularly when having to produce structures that are not very frequently used in the input children receive, as is the case for multiple interrogatives. Furthermore, the finding that differences between the heritage and the monolingual children surface only in production, while similar patterns emerge in the two groups in the real-time comprehension of multiple interrogatives, also suggests that on-line methods can better reflect competence in HL (see Villegas (2014) for similar results with adult heritage speakers).

An interesting observation here is the fact that children’s production patterns mirror the errors that they produce in the comprehension task. For example, reversed role errors are linked to children’s preference to mainly produce questions with a subject-object order for the conditions containing *which*-elements. Their production of simple wh-questions mirrors the list answers they give in the comprehension task. To recall, these were answers in which children answered only one wh-word (either by listing all the Agent characters in the image or by listing the Patient characters), indicating that they treat multiple wh-questions as simple questions. These errors in comprehension, together with children’s production patterns, show that even monolingual Romanian-speaking children take longer to produce structures with multiple wh-fronting and that they also make use of the option of having one wh-fronted and one *in-situ*. This corroborates Grebenyova’s (2011) findings for Russian as she shows that monolingual Russian-speaking children 4 to 6 years of age also have more difficulties with the production of multiple wh-questions compared to monolingual English-speaking children of the same age. She postulates that Russian-speaking children go through an intermediate phase when acquiring the syntax of multiple interrogatives in Russian and that, with enough Russian input, they will produce questions with multiple wh-fronting, similarly to adults. Although it is not very clear what would count as “enough Russian input” given that children rarely hear such multiple wh-questions to begin with, this view has interesting implications for our study as it suggests that, while monolingual Romanian children will eventually converge on the correct production of multiple wh-fronting questions, for the Romanian heritage children this will depend on the amount of Romanian input they receive. Future research with Romanian-dominant bilinguals and with Romanian heritage adults can shed light on this.

## Conclusion and future research

Our study makes a substantial contribution to the understanding of child HL development, as it investigates the production and comprehension of the same phenomenon in HL speakers and provides insight into the language development stages of both monolingual and heritage bilingual children. The current study is among the first to investigate cross-linguistic influence in bilingual children during real-time sentence processing and the first to use the self-paced listening paradigm with heritage children. However, the study also paves the way to future questions that remain unaddressed, such as when the syntax of multiple interrogatives is fully acquired as well what happens with adult heritage speakers or child heritage speakers whose dominant language(s) also have multiple wh-movement or lack this type of questions entirely. Studies that compare multiple bilingual groups will further increase our understanding of the impact of cross-linguistic influence on heritage language development.

## Data availability statement

The raw data supporting the conclusions of this article will be made available by the authors, without undue reservation.

## Ethics statement

The studies involving human participants were reviewed and approved by Ethics Committee of the University of Reading. Written informed consent to participate in this study was provided by the participants’ legal guardian/next of kin.

## Author contributions

AB carried out the data collection and was responsible for data analysis. AB and TM shared responsibility for the conception of the work and the interpretation of results. AB wrote the manuscript with input from TM. All authors contributed to the article and approved the submitted version.

## Funding

This research was supported by an Early Postdoc. Mobility grant for AB from the Swiss National Science Foundation (grant number 174870).

## Conflict of interest

The authors declare that the research was conducted in the absence of any commercial or financial relationships that could be construed as a potential conflict of interest.

## Publisher’s note

All claims expressed in this article are solely those of the authors and do not necessarily represent those of their affiliated organizations, or those of the publisher, the editors and the reviewers. Any product that may be evaluated in this article, or claim that may be made by its manufacturer, is not guaranteed or endorsed by the publisher.

## Footnotes

^1^The monolingual group (n=30) overlaps with the sample of children (n=32) reported in Bentea and Marinis (2021) which compared the online processing of multiple wh-questions in Romanian monolingual children and adults. In the current study, we investigated in addition the production of multiple interrogatives.
